# Infection with Kaposi's sarcoma-associated herpesvirus (KSHV) and human immunodeficiency virus (HIV) in relation to the risk and clinical presentation of Kaposi's sarcoma in Uganda

**DOI:** 10.1038/sj.bjc.6601113

**Published:** 2003-07-29

**Authors:** R Newton, J Ziegler, D Bourboulia, D Casabonne, V Beral, E Mbidde, L Carpenter, D M Parkin, H Wabinga, S Mbulaiteye, H Jaffe, R Weiss, C Boshoff, The Uganda Kaposi's Sarcoma Study Group

**Affiliations:** 1 Cancer Research UK, Epidemiology Unit, Gibson Building, Radcliffe Infirmary, Oxford OX2 6HE, UK; 2 Uganda Cancer Institute and Makerere University Medical School, Kampala, Uganda; 3 Cancer Research UK, Viral Oncology Group, Wolfson Institute of Medical Sciences, Cruciform Building, Gower Street, London WC1E 6BT, UK; 4 MRC Programme on AIDS, Uganda Virus Research Institute, PO Box 49, Entebbe, Uganda; 5 International Agency for Research on Cancer, 150 Cours Albert Thomas, Lyon, France; 6 Centers for Disease Control and Prevention, 1600 Clifton Road, Atlanta, Georgia, 30333, USA; 7 Windeyer Institute, University College London, 46 Cleveland Street, London, UK

**Keywords:** KSHV/HHV-8, Kaposi's sarcoma, HIV, Uganda

## Abstract

A case–control study from Uganda found that the risk of Kaposi's sarcoma increased with increasing titre of antibodies against Kaposi's sarcoma-associated herpesvirus (KSHV) latent nuclear antigens, independently of HIV infection. Clinically, widespread Kaposi's sarcoma was more frequent among patients with HIV infection than in those without, but was not related to anti-KSHV antibody titres.

The work described here was part of a large epidemiological study of cancer in Uganda (Newton *et al*, 2001, 2002). The findings in relation to Kaposi's sarcoma in HIV-seronegative and seropositive people and the seroepidemiology of Kaposi's sarcoma-associated herpesvirus (KSHV; human herpesvirus-8 (HHV-8)) are reported elsewhere (Ziegler *et al*, 1997, 2003; Newton *et al*, 2003). We examine here the role of antibodies against KSHV latent antigens encoded by orf73 and HIV serostatus on the clinical features and risk of Kaposi's sarcoma in Ugandan adults. Some of the results relating to anti-KSHV antibody titres and Kaposi's sarcoma in HIV-seronegative people have been published elsewhere (Ziegler *et al*, 2003).

## MATERIALS AND METHODS

Between 1994 and 1998, we recruited adults 15 years or older with a new diagnosis of cancer from the wards and outpatient clinics of the main hospitals in Kampala, Uganda. After informed consent and counselling, patients were interviewed and tested for infection with HIV-1 using the Cambridge Bioscience Recombigen ELISA (Cambridge, MA, USA) on sera or the GACELISA method (Murex, Dartford, UK) on saliva. The study was approved by the Committee on Human Research (VA Medical Centre and University of California San Francisco) and by the Uganda National Council for Science and Technology.

In total, we recruited 669 cases with Kaposi's sarcoma. Of these, 49% (331) had enough sera stored for anti-KSHV antibody testing and 77% (518) were seen by a study doctor, who conducted a clinical examination in which the physical location of tumour lesions was recorded. Where detected, the presence of internal lesions was also recorded. Cases were categorised according to whether the Kaposi's sarcoma lesions were clinically localised (i.e., restricted to just one area of the body, such as a limb, or an area of the torso or face), or widespread (i.e., affecting more than one area of the body). Cases with an internal lesion (e.g., oral, gastrointestinal or lung) were recorded as having widespread disease. For 297 cases with an assessment of the clinical stage of their disease at diagnosis enough sera were available to test for antiKSHV antibodies.

Controls comprised 306 people with non-malignant manifestations of HIV disease, recruited from the outpatient department of Mulago Hospital and 723 patients with other malignancies or benign tumours, for whom a stored blood sample was available for KSHV testing. The latter group comprised people with cancers of the oral cavity (28), oesophagus (37), stomach (20), liver (47), skin (23), breast (68), cervix (180), ovary (20), prostate (11), penis (13), eye (54), and non-Hodgkin's lymphoma (44), Hodgkin's disease (24), other cancer sites or types (125) and benign tumours (29).

A single investigator (DB) performed the immunofluorescence assays to detect antibodies against the latent nuclear antigen of KSHV encoded by orf73 and was blinded to the case–control status of the samples. Results were graded by fluorescent intensity and are reported here as negative (<1 : 100), one plus, two plus and three plus, indicating absent, weak, moderate and strong fluorescent signals, respectively. The fluorescent signal intensity was related to anti-KSHV antibody titre–the median titres were 1 : 800 for low-signal intensity (+), 1 : 25 600 for medium-signal intensity (++) and 1 : 51200 for high-signal intensity (+++). Further details of the testing procedure and scoring mechanism for signal intensity are described elsewhere (Sitas *et al*, 1999).

Data were computerised by trained clerks using EPI-INFO software and statistical analyses were conducted using STATA (Dean *et al*, 1990; STATA, 2001). Odds ratios (ORs) were estimated using unconditional logistic regression modelling with adjustment for age group (<30, 30–44, 45+) and sex. Tests for association used the *χ*^2^ test on one degree of freedom (unless otherwise stated). All *P*-values are two-sided.

## RESULTS

The proportion of Kaposi's sarcoma cases with widespread rather than localised disease at diagnosis was higher in HIV-infected than uninfected people (78% (337 out of 433) *vs* 36% (31 out of 85); *χ*^2^ (one degree of freedom (d. f.))= 32.9, *P*<0.001), but did not differ according to the fluorescent signal intensity of anti-KSHV antibodies either in HIV-seronegative (*χ*^2^ for trend=3.7, *P*=0.3) or in HIV-seropositive people (*χ*^2^ for trend=2.1, *P*=0.6; Table 1

**Table 1:** Distribution of anti-KSHV antibodies among those with Kaposi's sarcoma according to whether the patient had clinically widespread disease or not,^a^ stratified by HIV serostatus

	**Clinically widespread disease (percentage/number)**	
	**No**	**Yes**
HIV seronegative		
KSHV seronegative	50% (6)	50% (6)
KSHV seropositive	57% (30)	43% (23)
+	70% (14)	30% (6)
++	66% (12)	34% (6)
+++	44% (4)	56% (5)
		
HIV seropositive		
KSHV seronegative	31% (13)	69% (29)
KSHV seropositive	22% (43)	78% (151)
+	18% (16)	82% (71)
++	25% (22)	75% (65)
+++	25% (5)	75% (15)

a A patient was defined as having clinically widespread disease if lesions occurred in more than one area of the skin or if an internal (e.g., oral, lung, gastrointestinal tract) lesion was present.

).

Among controls, the seroprevalence of anti-KSHV antibodies was 46% (476 out of 1029) and did not vary significantly between the constituent subgroups of cancer sites or types and other diseases (*χ*^2^ for heterogeneity (14 d.f.)=21.9, *P*=0.1; data not shown). Anti-KSHV antibodies were present in 50% (302 out of 607) of HIV-seronegative controls and 41% (174/422) of HIV-seropositive controls, but after adjustment for age and sex, there was no significant association between infection with HIV and the presence of anti-KSHV antibodies (*χ*^2^ (1d.f.)=1.4, *P*=0.2). Nor was there any evidence that the fluorescent signal intensity, a measure of the titre of anti-KSHV antibodies, differed significantly between HIV-seropositive and -seronegative controls (*χ*^2^ for trend=2.3, *P*=0.5; Table 2

**Table 2:** Distribution of anti-KSHV antibodies among controls, stratified by HIV serostatus

	**HIV seronegative**	**HIV seropositive**
KSHV seronegative	50% (305)	59% (248)
		
KSHV seropositive		
+	28% (167)	27% (114)
++	17% (105)	11% (46)
+++	5% (30)	3% (14)
		
Total	100% (607)	100% (422)
		*χ*^2^ trend (1 d.f.)=2.3, *P*=0.5^a^

a Adjusted for age and sex; d.f.=degrees of freedom.

).

The seroprevalence of anti-KSHV antibodies among patients with Kaposi's sarcoma, was 81% (269 out of 331) and did not differ significantly between those with and without concurrent HIV infection (79% in HIV-seronegative *vs* 82% in -seropositive cases; *χ*^2^ (1d.f.)=1.8, *P*=0.2). The OR for Kaposi's sarcoma associated with anti-KSHV antibodies was 3.8 in HIV-seronegative people (95% confidence interval (CI) 2.1–6.8; *P*<0.001) and 6.3 in HIV-seropositive people (95% CI 4.2–9.2; *P*<0.001). The risk increased with increasing fluorescent signal intensity to anti-KSHV antibodies both in HIV-seronegative (*χ*^2^ for trend=29.7, *P*<0.001) and -seropositive people (*χ*^2^ for trend=106.1, *P*<0.001; Table 3

**Table 3:** Odds ratio (OR) for Kaposi's sarcoma according to fluorescent signal intensity of anti-KSHV antibodies, stratified by HIV serostatus

	**Cases**	**Controls**	**OR (95% CI) by titre**
HIV Seronegative			
KSHV seronegative	17	305	1.0
KSHV seropositive			
+	24	167	2.3 (1.2–4.7)
++	27	105	5.7 (2.8–11.6)
+++	12	30	7.1 (2.8–17.8)
	*χ*^2^trend (1d.f.)=29.7, *P*<0.001		
HIV Seropositive			
KSHV seronegative	45	248	1.0
KSHV seropositive			
+	88	114	4.1 (2.6–6.3)
++	95	46	10.8 (6.6–17.8)
+++	23	14	9.3 (4.3–20.2)
	*χ*^2^trend (1d.f.)=106.1, *P*<0.001		

ORs adjusted for age and sex; d.f.=degrees of freedom.

).

## DISCUSSION

Here, we show that the relative risk of Kaposi's sarcoma increased with increasing titre of antibodies against KSHV latent nuclear antigens among both HIV-infected and-uninfected individuals. The risk of Kaposi's sarcoma is increased in people with HIV infection and the disease tends to be more widespread and aggressive than in those without (Buchbinder and Friedman-Kien, 1991). However, in these data, HIV infection is not related either to the prevalence or to the titre of antibodies against KSHV latent antigens. Furthermore, among people diagnosed with Kaposi's sarcoma, clinically widespread disease was associated with HIV infection, but was not related to anti-KSHV antibody titres. We cannot exclude the possibility of some misclassification of tumour burden and the impact of this on our results is unclear. Nonetheless, on the basis of these data, the effect of HIV infection both on the risk and on the clinical presentation of Kaposi's sarcoma would seem to be unrelated to antibodies against KSHV latent antigens.

In this study, we could not quantify the impact of HIV infection specifically on the risk of Kaposi's sarcoma because a special emphasis had been placed on recruiting HIV-seronegative cases (Ziegler *et al*, 2003). However, results presented here in relation to KSHV antibody titres are in accord with those reported by Sitas *et al* (1999) from a case – control study of black South African cancer patients. Furthermore, in a cohort study of HIV-infected men in Europe, Rezza *et al* (1999) showed that high titres of anti-KSHV antibodies were predictive of the subsequent development of Kaposi's sarcoma among KSHV-infected individuals. These prospective data, together with the fact that the clinical burden of tumour lesions is independent of the titre of anti-KSHV antibodies (Table 1), argue against the possibility that high titres of antibodies against KSHV latent nuclear antigens result from the presence of Kaposi's sarcoma lesions. Presumably, the titre of antibodies against KSHV reflects the number of circulating KSHV-infected cells (i.e., the KSHV viral load), and that for a given titre, the burden of infected cells is higher among HIV-infected individuals than in those without HIV, and there is currently some evidence to support this (Tedeschi *et al*, 2001).

Little is known of the determinants of high antibody titres in individuals with anti-KSHV antibodies. In a previous report, we showed that among people with antibodies to KSHV in Uganda, the proportion with high titres increased with increasing age and with a past history of blood transfusion (Newton *et al*, 2003). Plancoulaine *et al* (2002) also showed that anti-KSHV antibody titres increased with age and were higher in men than in women. The determinants of high anti-KSHV antibody titres, their relationship with KSHV viral load and their role in the aetiology of Kaposi's sarcoma warrant further investigation.

## Appendix

The Uganda Kaposi's Sarcoma Study Group includes named authors and V Sembajwe, M Kalinaki, R Byansi, C Rwatooro, S Nambooze, B Tushemeirerwe, N Byabazaire (deceased), E Bitamazire, E Katabira, J Mugerwa (deceased), D Tindyebwa, C Ateenyi-Agaba, L Marum, J Whitworth, B Richardson, G Reeves and K de Cock.
